# The Dangers of Ignorance at Portsmouth

**Published:** 1912-03-09

**Authors:** 


### The Hospital

The Workers' Newspaper of Administrative Medicine and Institutional Life, Administration, National Insurance and Health.

No. 1339, Vol. LI. SATURDAY, MARCH 9th, 1912. PRICE ONE PENNY.

### THE DANGERS OF IGNORANCE AT PORTSMOUTH.

For several years the financial and general condition of the Royal Portsmouth Hospital has been going downhill. The hospital was established in 1849; it contains 146 beds; its total expenditure is over £9,000 a year, and its total ordinary income is under £7,000 a year. In 1907 annual subscriptions amounted to £2,764; in 1911 they had fallen to £2,682. In 1911 the amount received from the Hospital Sunday Fund was the lowest since 1887, with the result that there was a deficit of £1,967 on the year. By bringing in the whole of the extraordinary income this deficiency was reduced to £467. It would thus appear that the resources of this hospital have been diminishing since 1907, whilst the work and the number of in-patients have increased.

It is not a little remarkable, at a period in the history of voluntary hospitals when the public, and especially the working-classes, have shown an increasing disposition to add to their revenues, that the Royal Portsmouth Hospital should show a steady falling-off in its resources. It is fair to assume that the fault must lie to a great extent with the management, for policy has often much to do with popularity, and without popularity the hospital's resources must gradually be sapped. Our columns have shown from time to time that there have been grave differences of opinion in regard to this institution, and we have reason to think that the real cause of trouble will be found in the adoption of an unwise and narrow policy in the management of its affairs. This policy has, we understand, practically resulted in the control of the management falling into the hands of one section of the inhabitants, with the result that the larger and more fruitful field from which contributions and experience in the management of its affairs might have been secured has never been cultivated with success. We refer to the officers and professional men who form the larger portion of the population of Southsea. They have amongst them many distinguished officers of both Services who could, and we have no doubt would gladly give time and pecuniary help to the affairs of the Royal Portsmouth Hospital, if they were afforded an opportunity to do so without fear or favour.

The opinion we have just expressed seems to be justified by the proceedings at meetings of the Governors on January 5 and the 1st instant. At these meetings a determined attempt was made to change the rules which provide that the members of the honorary senior medical and surgical staff of the hospital shall not dispense or supply medicines or contract in any way for the supply of medicines. This is a necessary and general rule in regard to the staff of every hospital of importance throughout England. It is based upon the accepted rules which regulate the status of members of the medical profession according to their qualifications and work. We venture to say, for instance, that no practitioner is qualified to discharge adequately the responsible duties of a surgeon to a great hospital, unless he is freed from the burden of personal pharmacy and other necessary duties, which have to be discharged by practitioners whose patients are chiefly drawn from the humbler ranks of society. The enormous increase in surgical work, and in the responsibility of that work, and the need for highly trained and technical skill which every hospital surgeon worthy of the name must in these days possess, necessitate that no one who has not adequate time and special skill, based upon continuous practice, is eligible to discharge adequately the duties of surgeon, to an important modern hospital, like that at Portsmouth. We hope that the average of knowledge among the general public, certainly among the type of men from whom it is desirable that the larger proportion of members of each hospital committee should be drawn, is sufficient to enforce the truth and force of what we have just stated.

The discussion at the recent meetings of governors at Portsmouth proves, however, that there is a strong need there for the services of a trained administrator and man of influence and knowledge to go thoroughly, without a moment's delay, into the causes which lie at the root of the present shortness of funds at Portsmouth. It is certainly time that new blood should be brought into the management, when we find influential laymen on the committee urging, that, to enable practitioners who dispensed their own medicine, to be elected as physicians and surgeons to the hospital would not lower the standard of qualification for its honorary medical and surgical staff. A minister of religion is reported to have declared that the maintenance of a high standard for the honorary medical staff was merely to foster class distinctions which ought to be abolished. This view, to our surprise, seems to have been endorsed by the local Press, which is usually remarkable for the high standard of intelligence its editorial columns exhibit.

Another speaker asked whether a practitioner who supplied medicine thereby "lowered his ability, his brain power, and his social status." Certainly it necessarily lowers his professional status, and, whatever his ability and brain power may be, the drudgery of such work, and the time it absorbs, must prevent him from maintaining the skill and other qualifications essential to the discharge of the responsible duties of a modern surgeon. We are glad to see that the majority of the governors upheld the present rules, so that the Royal Portsmouth Hospital may still hope to secure the services of physicians and surgeons of the highest attainments. We have no doubt that the present managers of this hospital are interested in their work, but the views some of them expressed show that they are deficient in practical knowledge. It follows that, until the basis of representation upon the committee of this institution is wide enough and popular enough to include the leading representatives of all classes of the population, the day of its prosperity must linger, if it does not gradually disappear.

